# Informational needs in vulvar cancer and precursor conditions: a social listening study

**DOI:** 10.1007/s00520-026-11240-6

**Published:** 2026-09-22

**Authors:** D. Wouters, M. V. G. L. van Lynden van Sandenburg, K. K. Hulscher, E. M. G. van Esch, J. W. M. Aarts

**Affiliations:** 1 Amsterdam UMC, De Boelelaan 1117, Amsterdam, 1081 HV Netherlands; 2 Cancer Center Amsterdam - Treatment and Quality of Life (CCA), Amsterdam, Netherlands; 3 Amsterdam Reproduction & Development (AR&D), Amsterdam, Netherlands; 4 Catharina Ziekenhuis Eindhoven, Michelangelolaan 2, Eindhoven, 5623 EJ Netherlands; 5 Olijf Patiëntenorganisatie Gynaecologische Kanker, Utrecht, Netherlands; 6 Universiteit Van Amsterdam, Spui 21, Amsterdam, 1012 WX Netherlands

**Keywords:** Vulvar neoplasms, Social listening, Supportive care, Patient education, Health communication, Qualitative research

## Abstract

**Purpose:**

Vulvar cancer and precursor conditions including vulvar intraepithelial neoplasia (VIN) impose substantial burden, yet patient information provision remains poorly evidenced. Stigma and taboo contribute to diagnostic delays of 6–11 months. This study used social listening to map the online information-seeking landscape of women with vulvar cancer and VIN, with the aim of informing phase-integrated supportive care strategies.

**Methods:**

Questions were collected from six publicly accessible online platforms in the Netherlands, the UK, and the US (November 2025–January 2026), encompassing posts from 2008 onward. After screening 4859 posts, 619 questions were included. Data were analyzed using inductive qualitative content analysis with independent coding; reporting followed COREQ.

**Results:**

Seven themes were identified: Causes, Early signs, Diagnostics, Treatments, After treatment, Long-term concerns, and Psychosocial impact. Early signs and After treatment each accounted for 19% of coded segments, and Diagnostics for 17%. Recognition uncertainty—difficulty interpreting bodily changes as normal, abnormal, or recurrence-related—was the most frequently expressed informational need, accounting for 20% of all coded segments across the Early signs, After treatment, and Long-term concerns themes, more than any single theme. After treatment, questions predominantly concerned wound complications and recovery expectations, including among preoperative women.

**Conclusion:**

This recognition uncertainty is the central unmet need across the patient journey, from pre-diagnostic symptom appraisal through survivorship. These findings support phase-integrated supportive care interventions: initiating symptom-related communication before diagnosis, structuring preoperative education around recovery and wound care, and developing visually supported materials. Proactive clinician-patient communication could reduce diagnostic delays and psychological burden.

**Supplementary information:**

The online version contains supplementary material available at https://doi.org/10.1007/s00520-026-11240-6.

## Introduction

Despite the substantial physical, psychological, and social burden of vulvar cancer and its precursor conditions [1]—including vulvar intraepithelial neoplasia (VIN)—the supportive care and informational needs of affected women remain poorly characterized. The intimate nature of vulvar conditions contributes to stigma and social taboo, associated with feelings of shame, embarrassment, secrecy, and loneliness [2–5]. This taboo hinders symptom disclosure and timely healthcare seeking, prolonging uncertainty and emotional distress [2–6], and represents a significant barrier to delivering effective supportive care across the disease trajectory. These challenges highlight the importance of targeted, accessible patient information and communication strategies—both as components of supportive care and as means of reducing the burden associated with vulvar conditions.

Effective patient education and clinician-patient communication are central to reducing this burden. Patient information provides women with explanations of their condition, available treatment options, and potential consequences. However, patients across gynecological health conditions consistently report unmet informational needs, reflecting gaps in how information is communicated across the care pathway [6–12]. Research on informational needs in vulvar cancer and VIN has predominantly been conducted among women after diagnosis, focusing on experiences within clinical care pathways [1, 4, 13]. These largely qualitative studies have documented unmet communication needs regarding symptoms, precursor conditions, treatment, and post-operative care—but have not captured the substantial pre-diagnostic phase. A recent mixed-methods study similarly found that satisfaction with cancer information was lowest in the pre-diagnostic phase among women with vaginal and vulvar cancer, reinforcing the need to examine informational needs before formal diagnosis is established [14].

Online patient platforms and communities play a crucial role in shaping understanding, expectations, and decision-making, particularly when formal communication with healthcare providers has not yet been established [6, 8–11, 15]. In the pre-diagnostic phase, uncertainty may be heightened, and shame and fear continue to hinder open discussion of symptoms [1–5, 15]. Online platforms therefore offer a window into informational and communication needs that extend beyond those captured in clinic-based research.

Social listening—the systematic analysis of user-generated online content—enables identification of informational needs as they are spontaneously expressed, without researcher prompting [12, 16–18]. This method is particularly valuable for sensitive conditions where online environments allow articulation of concerns that might otherwise remain unspoken in clinical or research contexts [1–6, 8–11, 17, 18]. Prior studies in inflammatory bowel disease, metastatic breast cancer, and endometriosis have demonstrated that social listening can uncover underreported symptoms, unmet patient education needs, and gaps in existing educational resources [12, 16–18].

To date, no study has systematically examined population-level information-seeking or online communication needs related to vulvar cancer and its precursors. This study analyzed user-generated questions posted on Dutch, English, and American online platforms to identify knowledge gaps and unmet informational needs, with the aim of informing patient education and clinician-patient communication strategies.

## Methods

### Study design

**Fig. 1:**
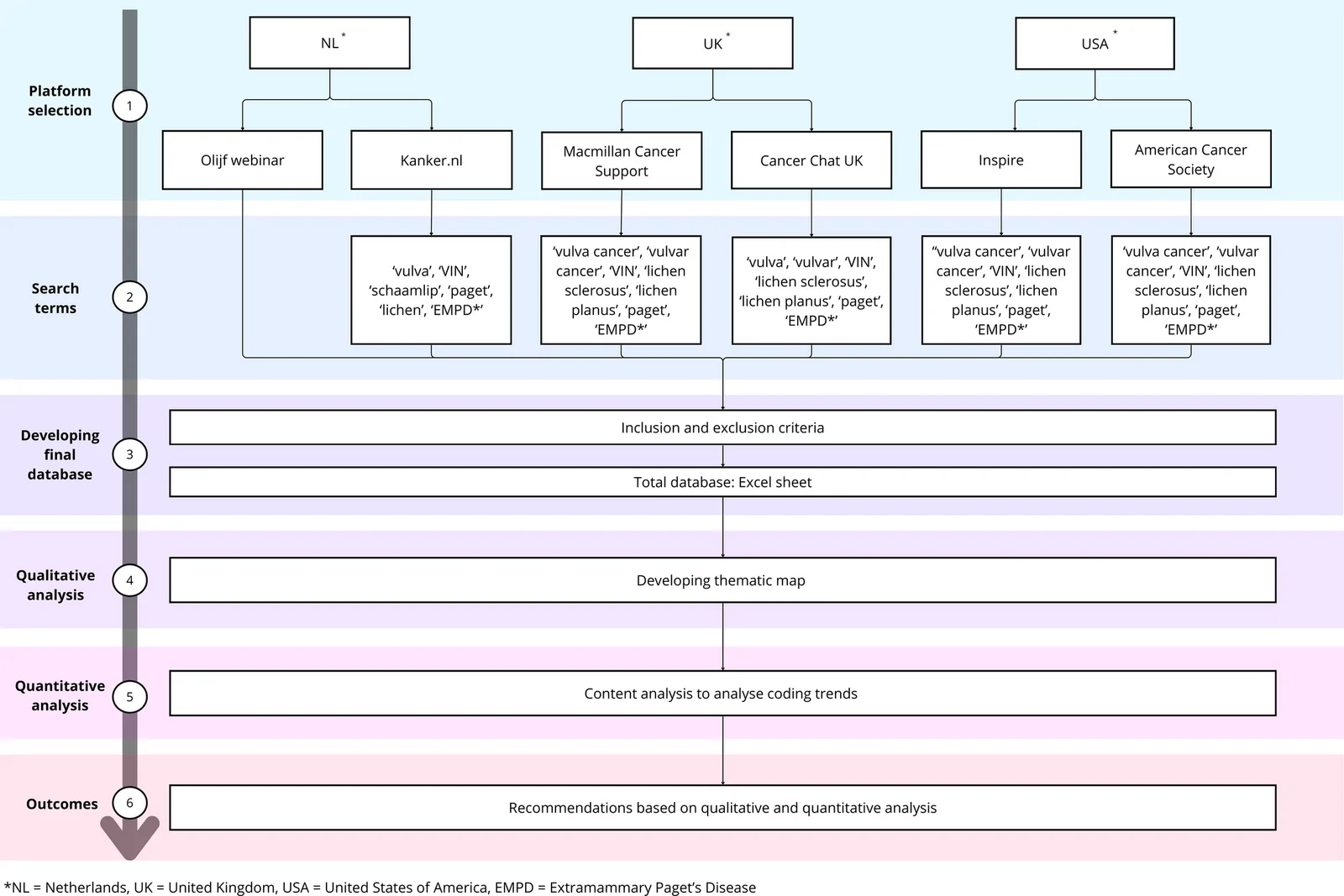
Six-step methodology overview

This study employed a social listening design, comprising passive identification, collection, and analysis of user-generated content from online sources. The methodological approach followed established social listening frameworks [12, 16, 17]. Data were collected from platforms in the Netherlands (NL), the United Kingdom (UK), and the United States (US) between November 2025 and January 2026. Reporting followed the Consolidated Criteria for Reporting Qualitative Research (COREQ) (see COREQ checklist, Multimedia Appendix A). An overview of the total six-step methodology is detailed in Fig. 1 and will be explained in greater depth below.

#### Step 1: Platform selection

Platforms were selected using predefined criteria: (1) health-specific platform with focus on gynecological conditions; (2) vulvar cancer or VIN content present; (3) publicly accessible without login requirements; (4) active within 2025–2026; and/or (5) referenced in national clinical guidelines or vulvar cancer care [19, 20]. Two platforms were selected per country. General social media platforms were excluded where health-related content could not be systematically retrieved. Selected platforms were:

- Netherlands: kanker.nl [21], the Dutch national online platform for cancer patients offering condition-specific discussion forums, and a recorded question-and-answer webinar hosted by patient association Olijf [22]
- UK: the Macmillan Cancer Support online community forum [23], and Cancer Chat UK [24], a moderated discussion forum hosted by Cancer Research UK
- US: Inspire health communities [25], a network of disease-specific online support communities, and the American Cancer Society Cancer Survivors Network [26], an online forum for patients and caregivers

Platforms were limited to the Netherlands, the UK, and the US based on the research team’s language proficiency (Dutch and English); posts were not translated into other languages, as translation risked altering the interpretation of nuanced symptom descriptions and expressed concerns. Country-level patterns were compared informally during analysis to assess whether findings reflected shared informational needs rather than nation-specific trends; no substantial differences in theme prevalence were observed across countries.

#### Step 2: Search strategy

Within each platform, predefined search terms related to vulvar cancer or its precursors were used to identify relevant posts (detailed in Fig. 1), adapted to platform-specific functionalities and language. No time limit was applied; the earliest post dated from 2008.

#### Step 3: Data collection

Questions were retrieved between November 2025 and January 2026. Posts were included if they contained a question related to vulvar conditions. All posts were read in full by the first author (DW); inclusion was not based on interrogative punctuation alone. A question was operationalized broadly as any post expressing an explicit or implicit informational need, including posts without interrogative phrasing that nonetheless conveyed an underlying request for information or reassurance (e.g., accounts of symptoms or treatment experiences implicitly seeking a response). Identification of implicit questions and the manual collection into an Excel dataset was performed by DW. Exclusion criteria were posts consisting solely of responses, duplicate questions from the same source, and posts containing no question. For the Olijf webinar transcript, questions posed during live sessions were extracted.

#### Step 4: Qualitative analysis

Data were analyzed using an inductive qualitative content analysis approach, allowing codes and themes to emerge directly from patient questions. All questions were manually reviewed during familiarization. Initial coding captured expressed informational needs, uncertainties, and requests for clarification. Codes were iteratively grouped and refined into overarching themes, defined here as manifest-level categories representing a shared informational-need domain across related codes, remaining close to the explicit content of the data rather than a latent, interpretive level of abstraction [27]; this manifest orientation reflects the nature of short, spontaneously generated questions, which offered limited scope for the deeper interpretive abstraction typically applied to in-depth interview data. To mitigate subjectivity from the data collection, all included posts were subsequently and independently coded in full by a second researcher (MLS), providing a check on the interpretation of both content and underlying intent. Discrepancies were resolved through discussion. Two gynecological oncologists reviewed preliminary themes to enhance clinical applicability (JA, EE).

#### Step 5: Quantitative analysis

Following theme development, descriptive frequency analysis was used to illustrate the relative prominence of themes [28, 29]. Frequencies were reported as proportions of total coded segments and used descriptively; as multiple codes could apply to a single question, cumulative percentages could exceed 100%.

#### Step 6: Outcomes and patient journey mapping

**Fig. 2:**
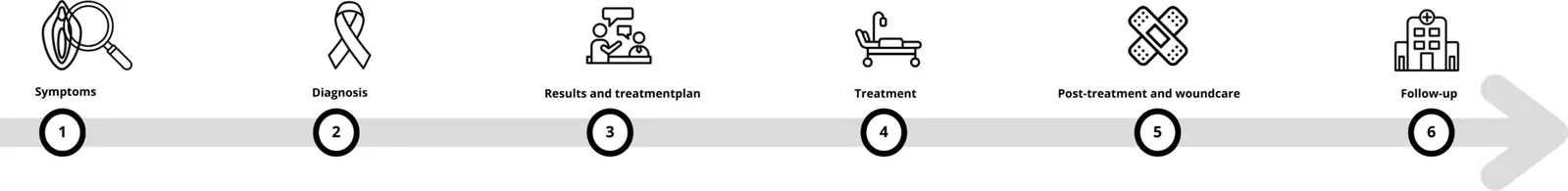
Patient journey

Based on thematic scope and relative prominence, practice-oriented recommendations were formulated to inform the development of patient-centered information materials and communication strategies (presented in “Discussion”). Themes were mapped onto a six-phase patient journey framework in Fig. 2: from initial symptom recognition (phase 1) through diagnosis, results, and treatment planning, treatment, post-treatment and wound care, to follow-up (phase 6). Final outcomes were discussed with an Olijf patient representative (KH), who has lived experience of gynecological cancer, to assess recognizability from a patient perspective; this built on an earlier presentation of preliminary findings to KH, which had similarly been confirmed as recognizable.

## Results

### Data collection

A total of 4859 posts were screened across six platforms in three countries, of which 619 questions met inclusion criteria (Multimedia Appendix B).

### Qualitative analysis: themes

**Fig. 3:**
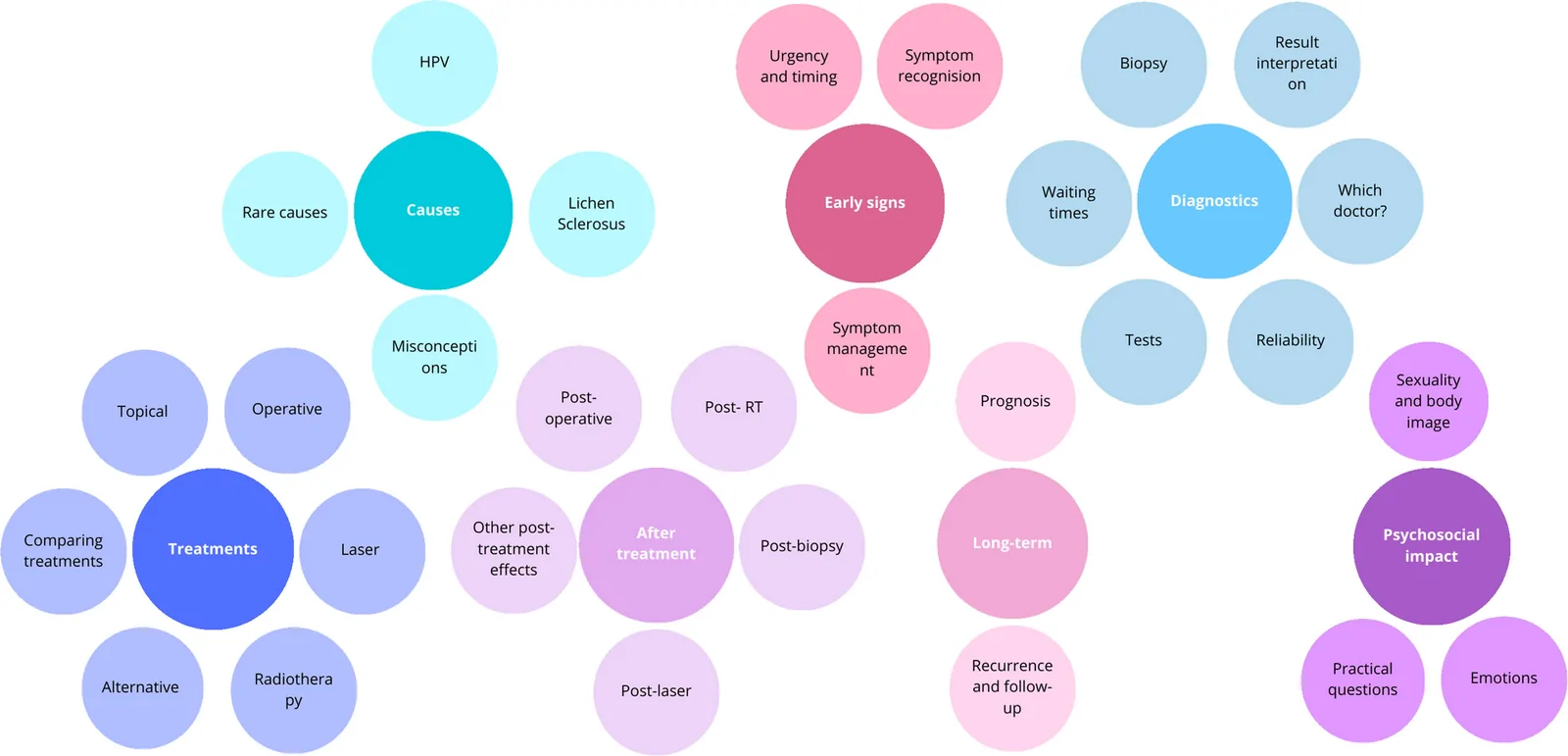
Thematic map

Content analysis identified seven overarching themes: causes, Early signs, Diagnostics, Treatments, After treatment, Long-term concerns, and Psychosocial impact, with associated subthemes (Fig. 3). Questions related to VIN and vulvar cancer were combined due to substantial overlap in symptoms, diagnostic pathways, and treatment approaches. All themes overlapped with key phases of the patient journey, with the exception of causes and psychosocial impact, which represented more overarching concerns.

### Quantitative analysis: theme distribution

The relative distribution of coded segments across themes is presented in Table 1. Early signs, after treatment, and diagnostics collectively accounted for the largest proportions (19%, 19%, and 17% respectively) and were identified as areas of highest expressed informational need.

**Table 1:** Relative distribution of coded segments per theme. *Three themes with highest expressed informational need

Theme	*n* (codes)	% of total codes
Causes	96	14
Early signs*	135	19*
Diagnostics*	122	17*
Treatments	93	13
After treatment*	131	19*
Long-term concerns	71	10
Psychosocial impact	55	8

### Causes

Within the causes theme, questions concerning lichen sclerosus (LS) and its relationship to vulvar cancer were most prevalent, followed by questions about HPV as a causal factor. A smaller number of questions addressed rarer causes and general misconceptions, including confusion about hereditary risk and the role of other skin conditions or infections in vulvar cancer development.

### Early signs: symptom recognition

Within Early signs, the symptom recognition subtheme dominated, accounting for 81% of codes within this theme and 16% of the total dataset—the single most frequently expressed informational need overall. The vast majority of posts (84%) combined descriptions of multiple concurrent symptoms with an explicit concern about vulvar cancer or VIN:

Got this spot that itches a bit, it’s just outside the vaginal opening on one side, a bit darker, and the tissue is different, like calloused, raised a little bit. Could this be vulvar cancer?

Vulva grey, sore and tender, a lot discharge. Worried I might have vulvar cancer or precancer.

The most commonly reported symptom triggers were lumps or nodules, followed by changes in skin color and persistent itching. A subset of posts explicitly requested visual information to support symptom interpretation, citing difficulty differentiating benign from concerning findings using existing online resources.

### Diagnostics: VIN terminology and result interpretation

Within diagnostics, result interpretation and tests were the most frequently coded subthemes, accounting for one-third and one-quarter of codes, respectively. Result interpretation questions predominantly concerned clarification of VIN terminology and its relationship to vulvar cancer:

I see people saying they are VIN 3 or VIN 2. What does this mean? Is this the cancer stage that they are in?

Is carcinoma in-situ the same thing as VIN 3?

Also I’m confused about the difference between having VIN 3 and vulvar cancer?

Questions within the tests subtheme focused on which diagnostic tests are used to identify vulvar cancer or its precursors. A smaller subtheme concerned waiting times, particularly regarding the duration biopsy results.

### Treatments

Within the treatments theme, questions most frequently concerned comparing treatment options and the use of complementary or alternative therapies. Operative and topical treatment questions were also common, while questions specific to laser therapy and radiotherapy occurred less frequently. Across subthemes, women frequently sought guidance on which treatment approach was most appropriate for their specific diagnosis and stage.

### After treatment: postoperative concerns

Within the after-treatment theme, postoperative questions accounted for half of all codes. These predominantly concerned uncertainty about whether experienced symptoms represented normal recovery or wound-related complications (29%), including wound infections, delayed healing, and lymphedema:

Anterior vulvectomy and lymph node removal. I am in so much pain and the swelling is unbelievable around my vulva. Is this normal?

Notably, 4% of the total dataset comprised postoperative questions from women who had not yet undergone surgery—more than twice as frequent as questions concerning the surgical procedure itself. A typical example illustrates this pattern:

I’m due to have surgery on Tuesday coming and was just wondering how people coped in their recoveries from this?

A further quarter of this theme concerned experiences following radiotherapy, focusing on late-onset symptoms including skin changes, adhesions, bladder and bowel problems, and chronic pain.

### Long-term concerns

Within the long-term concerns theme, questions were divided between recurrence and follow-up on one hand, and prognosis on the other, with recurrence and follow-up slightly more prevalent. Recurrence-related questions frequently expressed uncertainty about whether new or changing symptoms represented disease recurrence:

I’m noticing a lot of the area around the incision site (I had 12 stitches) is turning white like the tissue he removed. What does this mean, is the VIN spreading?

Prognosis-related questions concerned expected disease course and likelihood of cure, often in the context of long-standing or recurrent VIN.

### Psychosocial impact

Although psychosocial impact was not among the three most frequently coded themes, emotional expression was observed recurrently across posts. As coding was restricted to explicit questions, posts containing emotional content without a corresponding question were not counted within thematic frequencies, likely leading to underrepresentation of psychosocial burden. Within the symptom Recognition uncertainty subtheme, 30% of posts contained expressions of anxiety, worry, uncertainty, loneliness, or feeling overwhelmed. Questions within the Sexuality and body image subtheme concerned pain during intercourse, perceived anatomical changes, and sexual functioning after surgery or radiotherapy:

I’m worried what my vulva will look like after surgery because it’s not going to be symmetrical. I’m worried that I will be abnormal and no one will want me and I won’t be feminine anymore.

### Patient journey mapping

**Fig. 4:**
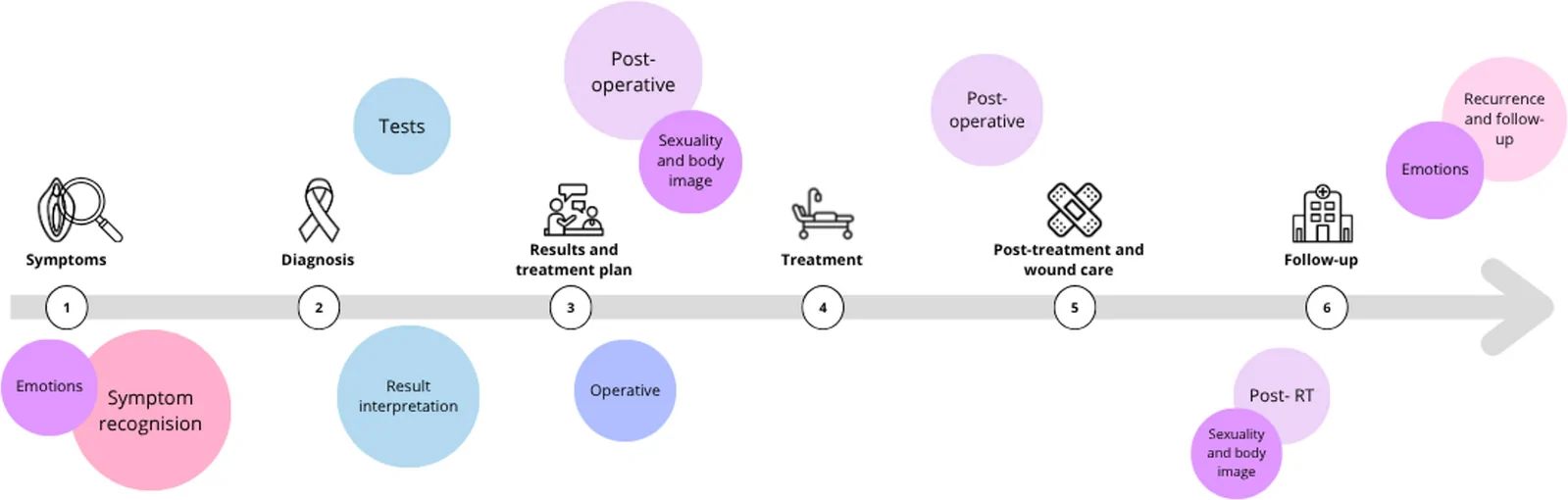
Theme correlation to patient journey

Figure 4 illustrates the distribution of themes across the six-phase patient journey. Informational needs were not confined to single phases: recognition uncertainty and psychosocial concerns recurred from pre-diagnostic symptom appraisal through survivorship, and peaked before formal diagnosis. Sexuality and body image concerns recurred from the treatment phase through follow-up.

To further illustrate this cross-phase persistence, we quantified recognition-related codes—i.e., codes explicitly concerned with interpreting bodily changes as normal or abnormal—across the dataset. Collectively, these codes accounted for 141 of 703 total coded segments (20%), exceeding the total number of codes within any single theme, including early signs itself (135 codes; Table 1).

Table 2 shows the distribution of these 141 recognition-related codes across the three patient journey phases in which they occurred. The large majority (77%) concerned prediagnostic symptom appraisal, with smaller but persistent proportions concerning postoperative wound complication recognition (15%) and recurrence recognition during follow-up (8%), together illustrating that recognition uncertainty, while concentrated before diagnosis, occurs during the whole patient journey.

**Table 2:** Recognition uncertainty-related codes across the patient journey

Patient journey phase	Recognition-related subtheme	*n* (codes)	% of recognition-related codes
Phase 1—Symptoms	Symptom recognition (early signs)	109	77%
Phase 5—Post-treatment and wound care	Postoperative wound complication recognition	21	15%
Phase 6—Follow-up	Recurrence recognition	11	8%
Total		141	100%

## Discussion

### Principal results

Recognition uncertainty—the persistent difficulty interpreting vulvar bodily changes—emerged as the dominant unmet informational need across all phases of the patient journey, with symptom recognition alone accounting for 16% of the total dataset. Notably, one in six questions originated from women not yet diagnosed, a population largely inaccessible to clinic-based research, demonstrating that substantial information needs arise before any contact with a healthcare provider.

### Comparison with prior work

These findings align with and extend prior qualitative work. Senn et al. [4] documented how women struggled to interpret unfamiliar bodily changes, while Williams et al. [6] identified poor symptom knowledge and cultural taboo as key barriers to timely help-seeking. The present study extends these observations by demonstrating the scale of recognition uncertainty at the population level without researcher prompting, capturing needs that clinic-based studies, limited to women already in care, cannot reach [4, 6, 30]. This gap is compounded by evidence of diagnostic delay in vulvar disease, including calls for clinical guidance on the early recognition of VIN [31], prolonged intervals from symptom onset to diagnosis [32], and gaps between referral and final diagnosis in specialized centers [33]. Postoperative informational needs align with clinical evidence that wound complications affect nearly half of women after vulvar surgery [34, 35]; that these questions were also posed by preoperative women points to a gap in perioperative communication not previously identified through clinic-based methods.

### Implications for patient communication and education

The identification of recognition uncertainty as a continuous, cross-phase unmet need challenges diagnosis-centric communication models that concentrate educational resources at the point of treatment. Patient education should begin before diagnosis, with phase-specific content integrated across the care trajectory. Recurrent requests for visual information indicate that format is as meaningful as content: illustrated symptom guides, self-examination resources, and postoperative healing timelines represent concrete development priorities [36, 37]. Structured preoperative consultations addressing recovery expectations and signs of complications would directly address the anticipatory information needs observed in this study. The cross-phase persistence of psychosocial concerns—including sexuality, body image, and fear of recurrence—further underscores the need for longitudinal communication strategies that extend beyond treatment completion.

Reaching women before diagnosis requires strategies beyond the clinical encounter. Reducing the stigma and taboo surrounding vulvar symptoms—for example, through public awareness campaigns—may lower the threshold for symptom disclosure and information-seeking among women not yet in contact with a healthcare provider. Current HPV-related public health messaging predominantly emphasizes cervical cancer prevention; extending this messaging to explicitly address vulvar cancer and VIN risk may improve early symptom recognition among women exposed to HPV. Ensuring that accurate, accessible information is readily available online is similarly important, given that online platforms were the primary source of the pre-diagnostic questions captured in this study.

### Strengths and limitations

The social listening design captures spontaneously expressed needs without researcher prompting and provides access to a pre-diagnostic population inaccessible to clinic-based research. Independent coding, clinical review by two gynecological oncologists, patient representative input, and COREQ-guided reporting enhance credibility and transferability. Limitations include the self-selected, digitally literate sample, which limits generalizability. Temporal heterogeneity across the 2008–2026 collection window may affect the relevance of older posts. The descriptive design precludes causal inference; frequencies reflect relative prominence within this dataset, not population prevalence. Restriction of coding to explicit questions likely underrepresents psychosocial burden [1]. VIN and vulvar cancer were not coded as separate categories, given substantial symptom and pathway overlap; the majority of coded questions concerned vulvar cancer specifically. Although implicit questions were included to capture indirectly expressed needs, more subtle forms of masked disclosure—such as hedging language or humor used to minimize symptoms—were not systematically captured and may represent an additional source of underrepresentation, particularly for psychosocial burden. Finally, the absence of user demographic data precludes subgroup analysis.

### Future research

Future research should triangulate these findings with clinic-based assessments to characterize convergence and divergence between online-expressed and clinically identified informational needs. Co-designed interventions—including visual symptom atlases, interactive recovery guides, and recognition decision aids—warrant prospective evaluation for impact on diagnostic delay, anxiety, and help-seeking behavior.

## Conclusions

Analysis of 619 user-generated questions from online platforms in the Netherlands, the UK, and the US shows that current patient information provision for vulvar cancer and VIN does not adequately address the variety, timing, or format of women’s informational needs. Most concerns related to early symptom recognition, diagnostic testing and interpretation, and recovery after surgery or radiotherapy. The most persistent unmet need involved interpreting vulvar bodily changes—from recognizing initial symptoms through identifying wound complications and signs of recurrence. Collectively, this recognition uncertainty accounted for one in five (20%) of all coded questions, more than any single theme identified in this study.

These findings call for phase-integrated patient education and proactive clinician-patient communication strategies. Practically, this means (1) initiating symptom-related communication before diagnosis; (2) providing structured preoperative information addressing recovery and wound care; (3) developing visually supported educational materials; and (4) maintaining longitudinal communication that addresses psychosocial concerns, sexuality, and body image across the full care trajectory.

## Supplementary information

Below is the link to the electronic supplementary material.

ESM 1 (DOCX 32.2 KB)

## Data Availability

The dataset of user-generated questions is not publicly available, as individual posts may be identifiable in context. Data are available from the corresponding author (D. Wouters) upon reasonable request, subject to confirmation that sharing complies with the terms of service of source platforms.

## Abbreviations

VIN: Vulvar intraepithelial neoplasia
COREQ: Consolidated Criteria for Reporting Qualitative Research
