# Supplementary material for: Informational needs in vulvar cancer and precursor conditions: a social listening study
Source: Support Care Cancer. 2026 Sep 22;34(10):1001. doi: 10.1007/s00520-026-11240-6 (PMC13597542; doi:10.1007/s00520-026-11240-6)
Supplement: Supplementary file 1 [file 520_2026_11240_MOESM1_ESM.docx]

# Multimedia Appendix A: COREQ Checklist

Consolidated Criteria for Reporting Qualitative Research (COREQ) — 32-item checklist

| **#** | **Item** | **Guiding question** | **Reported in this study** |
| --- | --- | --- | --- |
|  | **DOMAIN 1: Research team & reflexivity** |  |  |
| 1 | Interviewer / facilitator | Who conducted data collection? | D. Wouters (first author) |
| 2 | Credentials | What were the researchers' credentials? | D. Wouters: medical researcher (MD); J.W.M. Aarts and E.M.G. van Esch: gynecological oncologists (PhD); M.V.G.L. van Lynden van Sandenburg: independent coder (MD candidate) |
| 3 | Occupation | What was their occupation at the time of the study? | D. Wouters: PhD candidate; J.W.M. Aarts and E.M.G. van Esch: gynecological oncologists; K. Hulscher: patient representative (Olijf) |
| 4 | Gender | Was the researcher male or female? | All female |
| 5 | Experience & training | What experience did the researcher have? | D. Wouters: prior experience in qualitative health research; J.W.M. Aarts and E.M.G. van Esch: supervision of qualitative research in gynecological oncology |
| 6 | Relationship with participants | Was a relationship established prior to study commencement? | N/A |
| 7 | Participant knowledge of the researcher | Did participants know the researcher? | N/A |
| 8 | Interviewer characteristics | Were any characteristics reported that could influence findings? | N/A |
|  | **DOMAIN 2: Study design** |  |  |
| 9 | Methodological orientation | What theoretical framework informed the study? | Inductive qualitative content analysis |
| 10 | Sampling | How were participants selected? | Purposive |
| 11 | Method of approach | How were participants approached? | N/A |
| 12 | Sample size | How many questions/posts were included? | 619 questions (from 4,859 screened posts) |
| 13 | Non-participation | Were any posts excluded? Reasons? | 4,240 posts excluded: no question present, duplicate, or sole response to others |
| 14 | Setting | Where was data collected? | Workplace |
| 15 | Presence of non-participants | Were others present during data collection? | N/A |
| 16 | Description of sample | What were the characteristics of participants? | User demographics not available |
|  | **DOMAIN 3: Data collection** |  |  |
| 17 | Interview guide | Were questions, prompts, guides provided by the authors? Was it pilot tested? | N/A |
| 18 | Repeat interviews | Were repeat interviews carried out? If yes, how many? | N/A |
| 19 | Audio/visual recording | Did the research use audio or visual recording to collect the data? | No |
| 20 | Field notes | Were field notes made? | No |
| 21 | Duration | What was the duration of data collection? | Collection period: November 2025 - January 2026; posts from 2008 to 2026 |
| 22 | Data saturation | Was data saturation discussed? | N/A |
| 23 | Transcripts returned | Were transcripts returned to participants? | N/A |
|  | **DOMAIN 4: Analysis and findings** |  |  |
| 24 | Number of coders | How many people coded the data? | Two: D. Wouters (primary coder) and M.V.G.L. van Lynden van Sandenburg (independent coder) |
| 25 | Description of coding tree | Was a coding tree described? | Yes, thematic map with seven themes and associated subthemes (Figure 3) |
| 26 | Derivation of themes | Were themes derived inductively or deductively? | Primarily inductive |
| 27 | Software | What software was used for data management? | Microsoft Excel (data storage and organization); manual coding |
| 28 | Participant checking | Did participants provide feedback on the findings? | N/A |
| 29 | Quotations presented | Were participant quotations presented to illustrate the themes / findings? Was each quotation identified? | Yes, N/A |
| 30 | Data and findings consistent | Was there consistency between the data presented and the findings? | Yes |
| 31 | Clarity of major themes | Were major themes clearly presented in the findings? | Yes |
| 32 | Clarity of minor themes | Is there a description of diverse cases or discussion of minor themes? | Yes |

# Multimedia Appendix B: Search Outcomes

| **Country** | **Platform** | **Posts screened** | **Questions included** |
| --- | --- | --- | --- |
| Netherlands (total) |  | 432 | 149 |
|  | kanker.nl | 383 | 104 |
|  | Olijf webinar | 49 | 45 |
| United Kingdom (total) |  | 1810 | 289 |
|  | Macmillan Cancer Support | 1049 | 150 |
|  | Cancer Chat UK | 761 | 139 |
| United States (total) |  | 2617 | 181 |
|  | Inspire | 1589 | 103 |
|  | American Cancer Society | 1028 | 78 |
| Total |  | 4859 | 619 |
